# Effects of Tocilizumab in COVID-19 patients: a cohort study

**DOI:** 10.1186/s12879-020-05701-4

**Published:** 2020-12-22

**Authors:** Christine A. Vu, Kailynn J. DeRonde, Ana D. Vega, Meshell Maxam, Gregory Holt, Yoichiro Natori, Jose Gonzales Zamora, Veronica Salazar, Renata Boatwright, Stephen R. Morris, Daniela de Lima Corvino, Anmary Fernandez Betances, Leah Colucci, James Keegan, Andy Lopez, Andrew Hany Rezk, Yvette Rodriguez, Gabriela M. Moraru, Susanne Doblecki, David J. De La Zerda, Lilian M. Abbo

**Affiliations:** 1 Department of Pharmacy, Jackson Health System, Miami, FL USA; 2 Jackson Memorial Hospital, Pharmacy Services, B069, 1611 NW 12th Ave, Miami, FL 33136 USA; 3 Division of Pulmonary Critical Care, Department of Medicine, University of Miami Miller School of Medicine, Miami, FL USA; 4 Division of Infectious Diseases, Department of Medicine, University of Miami Miller School of Medicine, Miami, FL USA; 5 University of Miami Miller School of Medicine, Miami, FL USA; 6 Department of Pediatrics, Jackson Health System/Holtz Children’s Hospital, Miami, FL USA

**Keywords:** Coronavirus, COVID-19, Tocilizumab, Cytokine release syndrome

## Abstract

**Background:**

Due to the lack of proven therapies, we evaluated the effects of early administration of tocilizumab for COVID-19. By inhibition of the IL-6 receptor, tocilizumab may help to mitigate the hyperinflammatory response associated with progressive respiratory failure from SARS-CoV-2.

**Methods:**

A retrospective, observational study was conducted on hospitalized adults who received intravenous tocilizumab for COVID-19 between March 23, 2020 and April 10, 2020.

**Results:**

Most patients were male (66.7%), Hispanic (63.3%) or Black (23.3%), with a median age of 54 years. Tocilizumab was administered at a median of 8 days (range 1–21) after initial symptoms and 2 days (range 0–12) after hospital admission. Within 30 days from receiving tocilizumab, 36 patients (60.0%) demonstrated clinical improvement, 9 (15.0%) died, 33 (55.0%) were discharged alive, and 18 (30.0%) remained hospitalized. Successful extubation occurred in 13 out of 29 patients (44.8%). Infectious complications occurred in 16 patients (26.7%) at a median of 10.5 days. After tocilizumab was administered, there was a slight increase in PaO_2_/FiO_2_ and an initial reduction in CRP, but this effect was not sustained beyond day 10.

**Conclusions:**

Majority of patients demonstrated clinical improvement and were successfully discharged alive from the hospital after receiving tocilizumab. We observed a rebound effect with CRP, which may suggest the need for higher or subsequent doses to adequately manage cytokine storm. Based on our findings, we believe that tocilizumab may have a role in the early treatment of COVID-19, however larger randomized controlled studies are needed to confirm this.

**Supplementary Information:**

The online version contains supplementary material available at 10.1186/s12879-020-05701-4.

## Background

Coronavirus Disease 2019 (COVID-19) is a rapidly progressing disease with severe lung injury as the primary cause of death [1]. Lung autopsies have revealed histologic patterns of diffuse alveolar damage and perivascular T-cell infiltration in the presence of intracellular severe acute respiratory syndrome coronavirus 2 (SARS-CoV-2) [2]. Once invaded, the virus is known to cause immune dysfunction by activating various proinflammatory cytokines, resembling that of cytokine release syndrome (CRS) [3, 4]. Among the numerous cytokines that are released, interleukin-6 (IL-6) is thought to play a major role in causing acute respiratory distress syndrome (ARDS) [5, 6].

Tocilizumab, an antagonist of soluble IL-6 receptor, is being evaluated for the management of COVID-19. Previously approved for the treatment of severe or life-threatening chimeric antigen receptor (CAR) T cell-induced CRS, its ability to downregulate the immune system may reduce the  detrimental  effects of COVID-19 [7, 8]. Studies have demonstrated tocilizumab to be associated with improvements in inflammatory markers, clinical response, and survival [9–21]. During an unprecedented time when proven effective therapies are lacking, we aimed to describe our real-life experience using tocilizumab for COVID-19.

## Methods

### Setting

We retrospectively analyzed hospitalized patients who received intravenous (IV) tocilizumab for COVID-19 within our large health care system in Miami, Florida between March 23, 2020 and April 10, 2020. Our health system is comprised of three acute care facilities with over 2500 licensed beds, including 150 adult intensive care unit beds. This study was approved by the University of Miami Institutional Review Board and Jackson Health System Clinical Research Review Committee and a waiver of informed consent was granted.

### Tocilizumab process

Tocilizumab was restricted to the Antimicrobial Stewardship Program with pre-approval authorization for the management of highly suspected or laboratory-confirmed SARS CoV-2 infection. The approval consisted of a screening process to see if the patient first met criteria for tocilizumab, followed by a multidisciplinary discussion between the treating physician, infectious diseases physicians, pulmonary/critical care physicians, and pharmacists. Since February 2020, we followed an institution-specific clinical protocol to determine when to consider COVID-19 investigational agents**.** For tocilizumab, patients were eligible if they met all of the following criteria: requiring ≥4 liters of nasal cannula to maintain a SpO2 > 93%, demonstrate signs of clinical deterioration, have elevations in at least two inflammatory markers (interleukin-6 > 40 pg/ml, C-reactive protein > 10 mg/dL, lactate dehydrogenase > 350 U/L, ferritin > 1000 ng/mL, D-dimer > 1 mcg/ml). Tocilizumab was not recommended in patients with concominant bacterial infections, baseline ALT or AST > 5 times the upper limit of normal (ULN), baseline platelet count < 100 × 10^9^/L, baseline absolute neutrophil count < 1.5 × 10^9^/L, or known history of diverticular disease or gastrointestinal perforation. However, exceptions were made if the treating physician believed the potential benefit could outweigh the risk. We administered flat doses of 400 mg (30–100 kg) and 600 mg (> 100 kg) based on the limited evidence and resource allocations during that time [11, 22].

### Study participants

Eligible patients were hospitalized adults (age ≥ 18 years) with suspected or laboratory-confirmed SAR-CoV-2 infection and received at least one dose of IV tocilizumab. Any patients with high clinical suspicion for COVID-19 and later confirmed as negative by qualitative real-time PCR were excluded. All patients received standard of care treatment for COVID-19 based on our institution-specific protocol, which at the time included hydroxychloroquine. Other therapies such as methylprednisolone, intravenous immunoglobulin, and convalescent plasma were recommended on a case-by-case basis. Data to support dexamethasone for COVID-19 was published after the completion of our study.

### Outcomes and definitions

The electronic medical record was retrospectively reviewed to collect data on day − 1, 0, 1, 2, 3, 4, 5, 7, 10, 14 and 30 relative to tocilizumab administration. We recorded laboratory and respiratory parameters, clinical improvement (defined as ≥2-point reduction on the WHO COVID-19 ordinal scale), all-cause mortality, proportion of patients discharged, proportion of patients requiring oxygen support, proportion of patients requiring intensive care unit (ICU) care, proportion of patients successfully extubated (defined as not requiring re-intubation within the same hospitalization), and infectious complications within 30 days of receiving tocilizumab [23]. Infectious complications were defined as having a positive culture from a sterile site and treated by the medical team; we excluded cases of suspected colonization or contamination. Oxygenation was assessed by calculating PaO_2_/FiO_2_ from the morning arterial blood gas (ABG) and corresponding FiO_2_. For infrequent cases when an ABG was not available to measure the PaO_2_, we used an estimation formula based on the corresponding SpO2, S/F = 64 + 0.84 * PaO_2_/FiO_2_ [24]. Acute respiratory distress syndrome (ARDS) was defined according to the Berlin Criteria [25].

### Statistical analysis

Descriptive statistics were used to analyze our study population. Continuous variables were expressed as median and range while categorical variables were expressed as counts and percentages. Pearson’s chi-squared test or Fisher’s exact test (as appropriate) was used to compare categorical data. Mann-Whitney-Wilcoxon test was used to compare ordinal data. A *p*-value of < 0.05 was considered statistically significant. All statistics were performed using SPSS (version 24, Chicago, IL).

## Results

A total of 63 patients received tocilizumab during our study period **(see Additional file** **1****: Appendix 1 supplemental material)**. Three patients were excluded after they were empirically treated as “patients under investigation” per the US Centers for Disease Control and Prevention criteria and then later confirmed to have a negative qualitative real-time PCR with an alternative diagnosis of infection. Patient characteristics are described in Table 1. Most patients were male (66.7%), Hispanic (63.3%) or Black (23.3%), with a median age of 54 years old (range 26–87). The most common comorbidities were obesity (58.3%), hypertension (53.3%), and diabetes (25.0%). The median time from symptom onset to hospital admission was 6 days. The median time from hospital admission to receiving tocilizumab was 2 days. The median time from symptom onset to receiving tocilizumab was 8 days, and this did not differ between the subgroup of patients who died. A majority of patients received hydroxychloroquine (86.7%). Of the 32 patients that received steroids, 12.5% received < 6 mg/day of dexamethasone equivalents, 15.6% received 6–20 mg/day of dexamethasone equivalents, and 71.9% received > 20 mg/day of dexamethasone equivalents. The median weight for our cohort was 91.5 kg (range 59–182) and the average dose of tocilizumab administered was 4.75 mg/kg. Forty-seven patients received a flat dose of 400 mg and the remaining received 600 mg. Only 3 patients received a second dose of tocilizumab.

**Table 1:** Patient characteristics

	All (*N* = 60)	Died (*n* = 9)
Age, median (range), years	54 (26–87)	58 (33–84)
Male, *n* (%)	40 (66.7)	8 (88.9)
Ethnicity		
Hispanic	38 (63.3)	6 (66.7)
Black	14 (23.3)	2 (22.2)
White	7 (11.7)	1 (11.1)
Asian	1 (1.7)	0 (0.0)
Comorbidities		
Obese (BMI > 30)	35 (58.3)	6 (66.7)
Hypertension	32 (53.3)	8 (88.9)
Diabetes	15 (25.0)	3 (33.3)
Congestive heart failure	4 (6.7)	1 (11.1)
Coronary artery disease	1 (1.7)	0 (0.0)
Asthma	4 (6.7)	0 (0.0)
COPD	1 (1.7)	1 (11.1)
Obstructive sleep apnea	2 (3.3)	0 (0.0)
HIV	1 (1.7)	0 (0.0)
Transplant	1 (1.7)	0 (0.0)
Concomitant therapies		
Hydroxychloroquine	52 (86.7)	8 (88.9)
Corticosteroids	32 (53.3)	5 (55.6)
Inhaled nitric oxide	5 (8.3)	1 (11.1)
Intravenous immunoglobulin (IVIG)	4 (6.6)	0 (0.0)
Tacrolimus	2 (3.3)	1 (11.1)
Convalescent plasma	2 (3.3)	0 (0.0)
Plasmapheresis	1 (1.7)	0 (0.0)
Time from symptom onset to hospital admission, median (range), days	6 (1–14)	7 (1–14)
Time from hospital admission to receiving tocilizumab, median (range), days	2 (0–12)	1 (0–4)
Time from symptom onset to receiving tocilizumab, median (range), days	8 (1–21)	8 (1–15)

Note: abnormal medians highlighted in bold

^a^Luo H, et al. *Clin Lab* 2019;65(3).

The clinical presentation of patients on the day of tocilizumab administration are described in Table 2. For disease severity, most patients scored a 4 (40.0%) or 7 (28.3%) based on the WHO COVID-19 ordinal scale. Most patients received oxygen supplementation via nasal cannula (30.0%) or invasive mechanical ventilation (38.3%). The median PaO_2_/FiO_2_ was 166 (range 33–523) and 50 patients (83.3%) had ARDS. For abnormal laboratory values, we observed neutrophilia, lymphopenia, elevated neutrophil-to-lymphocyte ratio, elevated aspartate aminotransferase (AST), along with increased levels of interleukin-6 (IL-6), C-reactive protein (CRP), erythrocyte sedimentation rate (ESR), lactate dehydrogenase (LDH), ferritin, procalcitonin, D-dimer, and troponin.

**Table 2:** Clinical presentation on day of tocilizumab administration

Disease severity	*n* (%)		
WHO Ordinal Scale			
8 (deceased)	0 (0.0)		
7 (invasive mechanical ventilation + organ support)	17 (28.3)		
6 (invasive mechanical ventilation)	9 (15.0)		
5 (non-invasive ventilation or high-flow oxygen)	9 (15.0)		
4 (oxygen by mask or nasal prongs)	24 (40.0)		
3 (hospitalized without oxygen therapy)	1 (1.7)		
1–2 (not hospitalized)	0 (0.0)		
Temperature ≥ 38 °C	28 (46.7)		
Heart rate ≥ 100 beats/min	34 (56.7)		
Respiratory rate ≥ 30 breaths/min	36 (60.0)		
Abnormal chest imaging	59 (98.3)		
Vasopressor use	18 (30.0)		
Renal replacement therapy	4 (6.7)		
Use of paralytics	9 (15.0)		
Proned	5 (8.3)		
Room air	1 (1.7)		
Nasal cannula	18 (30.0)		
Venti-mask	3 (5.0)		
Nonrebreather	7 (11.7)		
High-flow nasal cannula	6 (10.0)		
Non-Invasive Positive Pressure Ventilation	2 (3.3)		
Invasive mechanical ventilation	23 (38.3)		
ARDS			
Mild (201 < PaO_2_/FiO_2_ ≤ 300)	13 (21.7)		
Moderate (101 < PaO_2_/FiO_2_ ≤ 200)	21 (35.0)		
Severe (PaO_2_/FiO_2_ ≤ 100)	16 (26.7)		
PaO_2_/FiO_2_, median (range)	166 (33–523)		
SOFA score, median (range)	3 (0–11)		
ICU care	45 (75.0)		
Laboratory parameters	Median (range)	Reference values	Number of patients with available data
White blood cell count, ×10^9^/L	9 (2.7–29.6)	4.0–10.5	51
Absolute neutrophil count, ×10^9^/L	**6.85 (1.8–26.8)**	2.0–6.0	49
Absolute lymphocyte count, ×10^9^/L	**0.8 (0.2–2.6)**	1.1–2.7	48
Neutrophil-to-lymphocyte ratio (NLR)	**7.56 (2.25–62)**	0.88-4^a^	48
Hemoglobin, g/dL	12.7 (9–15.9)	11.1–14.6	51
RDW-CV, %	14 (11.6–18.3)	11–15	51
Platelets, ×10^9^/L	240 (101–513)	140–400	49
Sodium, mmol/L	135 (123–148)	135–145	53
CO_2_, mmol/L	24 (11–36)	22–30	53
AST, U/L	**70.5 (25–711)**	15–46	46
ALT, U/L	51.5 (6–242)	9–52	46
Total bilirubin	0.65 (0.2–2.4)	0.2–1.3	48
Creatinine, mg/dL	0.88 (0.4–4.58)	0.66–1.25	53
Interleukin-6, pg/mL	**133.9 (8.73–2160.69)**	none	26
C-reactive protein, mg/dL	**24.2 (3.2–45)**	0.0–0.9	49
Erythrocyte sedimentation rate, mm/hr	**50 (18–102)**	0–10	24
Lactate dehydrogenase, U/L	**1333 (477–5089)**	313–618	47
Ferritin, ng/mL	**1412.5 (45–29,304)**	30–400	46
Procalcitonin, ng/mL	**0.40 (0.027–16.34)**	0–0.08	33
D-dimer, mcg/mL	**1.3 (0.4- > 20)**	0–0.49	33
Troponin, ng/mL	**0.104 (< 0.012–7.21)**	0–0.034	15

Outcomes for patients within 30 days from receiving tocilizumab are summarized in Table 3. A total of 36 patients (60.0%) achieved clinical improvement, 9 patients (15.0%) died, 33 patients (55.0%) were discharged from the hospital alive, and 18 patients (30.0%) remained hospitalized. Of those who clinically improved, 13 patients (36.1%) received concomitant steroids. A total of 52 patients (86.7%) resided in the ICU and 29 (48.3%) patients required invasive mechanical ventilation. Thirteen patients (44.8%) were successfully extubated within 30 days of tocilizumab administration. We identified 29 cultures in 16 patients (26.7%) who developed infectious complications post-tocilizumab, with 10 (62.5%) of these patients having received steroids. The median time to first infection was 10.5 days (range 2–28). The most common types of infection were respiratory (48.3%) and bloodstream (48.3%) **(see Additional file** **1****: Appendix 3 supplemental material). We describe additional clinical measures pertaining to organ complications, modes of ventilation, and SOFA scores in Additional file** **1**: **Appendix 2 supplemental material**.

**Table 3:** Outcomes within 30 days of receiving tocilizumab

*n* (%)	All	Received steroids	No steroids	*p*-value
**Clinical improvement**	**36/60 (60.0)**	13/36 (36.1%)	23/36 (63.9%)	0.0015
WHO ordinal scale on day of tocilizumab administration, median (range)	4 (3–7)	4 (4–7)	4 (3–7)	0.47
WHO ordinal scale on day 30, median (range)	1 (1–4)	1 (1–4)	1 (1–4)	-
**Mortality**	**9/60 (15.0)**	5/9 (55.6%)	4/9 (44.4%)	1.00
Time to death from receiving tocilizumab, median days (range)	6 (1–14)			
**Discharged alive**	**33/60 (55.0)**	15/33 (45.5%)	18/33 (54.5%)	0.27
Hospital length of stay, median days (range)	15 (0–32)			
**Required ICU care**	**52/60 (86.7)**	31/52 (59.6%)	21/52 (40.4%)	0.02
Remained admitted to ICU at day 30	13/52 (25.0)			
Step down to floor at day 30	5/52 (9.6)			
Discharged from hospital alive by day 30	25/52 (48.1)			
Died by day 30	9/52 (17.3)			
**Required invasive mechanical ventilation**	**29/60 (48.3)**	19/29 (65.5%)	10/29 (34.5%)	0.12
**Successful extubation**	**13/29 (44.8)**	7/13 (53.8%)	6/13 (46.2%)	0.27
Duration of mechanical ventilation, median days (range)	15 (6–35)			
**Infectious complications**	**16/60 (26.7)**	10/16 (62.5%)	6/16 (37.5%)	0.57
Time to first infection, median days (range)	10.5 (2–28)			
Cultures drawn while in ICU, *n* (%)	26/29 (89.7)			
Cultures drawn while intubated, *n* (%)	25/29 (86.2)			
Type of infection, *n* (%)				
Respiratory	14/29 (48.3)			
Bloodstream	14/29 (48.3)			
Urinary	1/29 (3.4)			

**Fig. 1:**
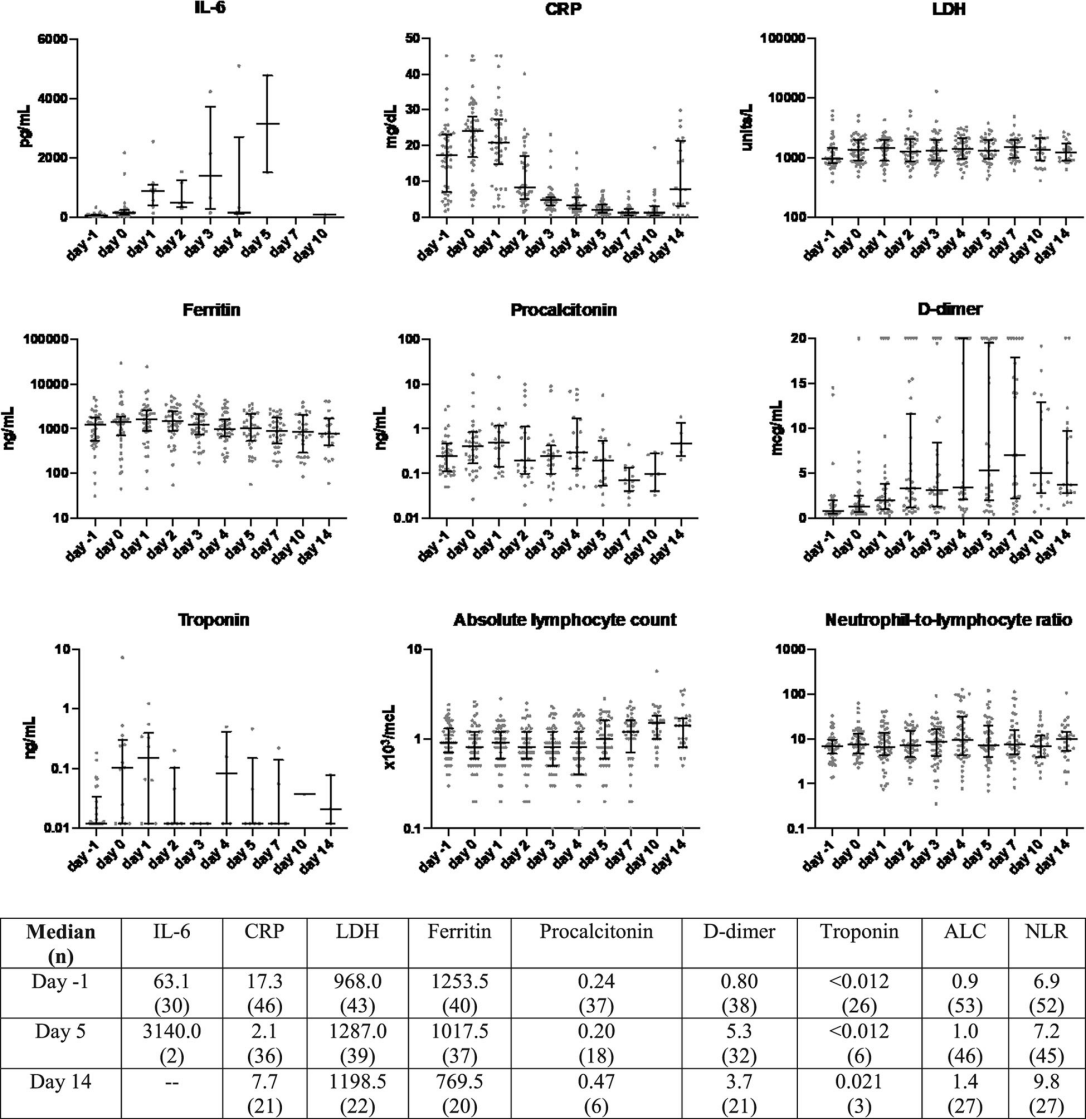
Progression of laboratory markers within 14 days of tocilizumab (results shown as median and IQR using Prism GraphPad version 8). Troponin: lower limit of detection < 0.012 ng/ml; D-dimer: upper limit of detection > 20 mcg/ml

**Fig. 2:**
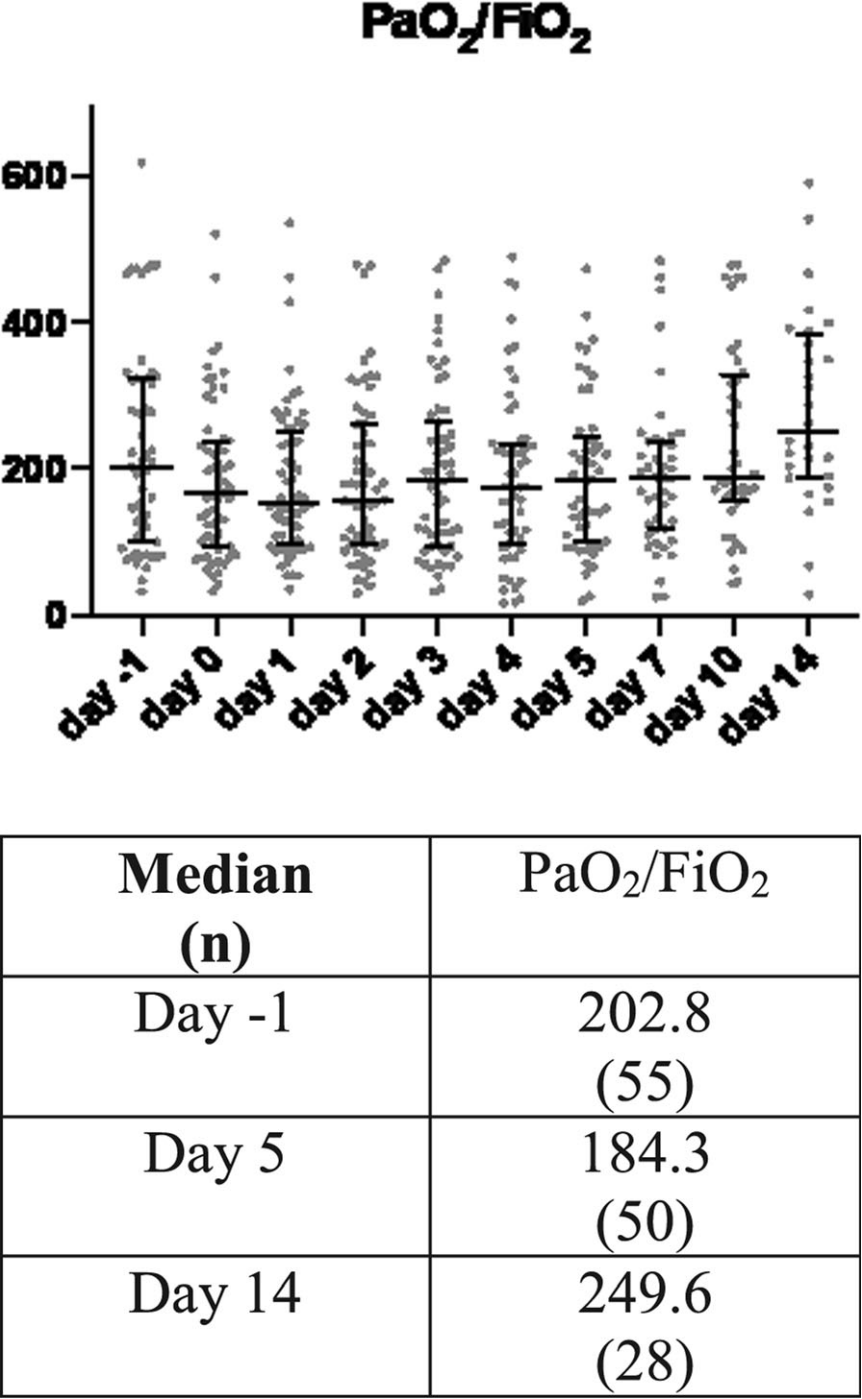
Progression of oxygenation within 14 days of tocilizumab (results shown as median and IQR using Prism GraphPad version 8)

The progression of select laboratory and respiratory parameters within 14 days of tocilizumab are displayed in Fig. 1 and Fig. 2. We observed an initial reduction in CRP; however levels began to rise again after day 10. The opposite effect was seen with D-dimer. We saw an increase in IL-6 and improvements in both lymphopenia and oxygenation as measured by PaO_2_/FiO_2_. No clear trends were seen for lactate dehydrogenase, procalcitonin, troponin, or neutrophil-to-lymphocyte ratio (NLR).

## Discussion

During the rapidly spreading pandemic, providers were faced with the challenge of recommending investigational agents for the treatment of COVID-19. Since elevated IL-6 levels have been associated with ICU admission, ARDS, and death, we chose to prescribe tocilizumab in patients with suspected CRS [6]. We aimed to provide early administration of tocilizumab in patients not yet on mechanical ventilation but with signs of worsening disease. Results from the EMPACTA trial demonstrated that patients who received tocilizumab were 44% less likely to progress to mechanical ventilation or death [26]. In our cohort, 9 out of the 31 patients who received tocilizumab early on later progressed to invasive mechanical ventilation. In the future, we hope to explore the effects of tocilizumab timing, as there may be a window of opportunity for preventing progressive respiratory failure.

Our patients presented with typical manifestations of COVID-19 and signs and symptoms of cytokine release syndrome. Similar to previous reports, patients with more severe disease demonstrated transaminitis, along with abnormal blood counts such as neutrophilia, lymphopenia, and elevated NLR ratio [4, 27]. After receipt of tocilizumab, CRP levels decreased but unlike other studies, this effect was not sustained [10–12]. CRP started to increase again after day 10, which correlates with tocilizumab’s elimination half-life of 11 to 13 days [28]. Compared to other studies where reductions in CRP remained until day 14, our patients received lower doses and the majority did not receive a second dose [14, 17]. This suggests that tocilizumab’s effect on CRP may be dose-dependent and that re-dosing after 10 days may be warranted. Previous pharmacokinetic data has also suggested that at least two doses of tocilizumab are needed to achieve adequate drug levels in plasma [7]. As expected, repeat IL-6 levels, although only available for one third of our patients, increased quickly after tocilizumab administration. This effect is known to occur after competitive binding of tocilizumab to the IL-6 receptor, resulting in the temporary accumulation of free IL-6 in the serum [29]. We also observed an increase in D-dimer that peaked at day seven, and then decreased. Some have correlated D-dimer with the risk of developing pulmonary embolism in COVID but this was not investigated in our study. No clear trends were seen for LDH or procalcitonin, suggesting that these markers are non-specific to COVID-19.

There are mixed results on the effects of oxygenation after tocilizumab administration in COVID-19 patients. Studies have reported improvements in oxygenation while others did not [11, 13, 30]. We observed an overall increase in PaO_2_/FiO_2_ after tocilizumab, but it is unclear whether this was a drug effect or more so reflects the natural course of ARDS. One study  found no association between tocilizumab and FiO_2_ reduction [17]. By day 30, extubation occurred in 13 out of 29 patients (44.8%). Rates of extubation for COVID-19 have only been recorded in a small study where 2 out of 3 patients were successfully extubated after receiving tocilizumab [10].

By our study endpoint, 36 patients (60.0%) demonstrated clinical improvement and 33 patients (55.0%) were discharged alive. Our discharge rate was very similar to the 56% reported by Somers et al. [15]. We observed a 30-day mortality rate of 15%, which falls within the range (13–27%) of previous studies [10, 12, 15, 16, 30, 31]. Many studies have already investigated the relationship of tocilizumab and mortality in COVID-19 patients, but with mixed findings. Salvarani et al. and Campochiaro et al. found no significant difference in mortality in patients receiving tocilizumab [18, 31]. In contrast, several other studies have shown tocilizumab to be associated with  a decreased risk of death, lower hospital-related mortality, as well as reduced risk of all-cause mortality [15–17, 19, 20]. However, it is important to note that many of these studies allowed for concominant steroid use, a known confounder towards better survival [32]. When comparing our patients who received steroids with tocilizumab to those who did not, the cohort who received steroids surprisingly did worse. More patients died, less demonstrated clinical improvement, and less were discharged from the hospital alive despite having similar baseline COVID-19 disease severity. We believe this could be explained by the use of  higher steroid  doses in our study; most patients received dosages greater than 6 mg daily of dexamethasone equivalents and as a result,  were more  immunosuppressed than the RECOVERY population.

Another hypothesis for worse outcomes when combining steroid with tocilizumab is the higher incidence of infectious complications in the steroid group (62.5% vs. 37.5%, *p* = 0.57), although this was not statistically significant. Tocilizumab is immunosuppressive and has been linked to secondary infections [33, 34]. In our study, we identified an overall infection rate of 26.7% within 30 days of receiving tocilizumab. Another study with a longer follow-up time of 8 weeks found a higher infection of incidence at 64.2%; however, they used a broader definition for infections that included both  highly suspected infections and confirmed infections [35]. Additional studies have reported infection rates possibly secondary to tocilizumab [15–17]. Somers et al. reported a two-fold higher incidence of infection (54% vs. 26%, *p* < 0.001) however more patients in the tocilizumab arm received steroids [15]. So far, the only study that excluded concomitant steroids found a lower incidence of infections with tocilizumab (8.1% vs. 17.3%, *p* = 0.03) [21]. Therefore, it remains unclear whether tocilizumab, when used by itself, increases the risk of infection.

Our study had several limitations. First, it was a small retrospective study with no matched control group. Second, the flat doses of 400 and 600 mg for tocilizumab could have resulted in lower than optimal doses if extrapolating from FDA-approved (8 mg/kg) doses for CAR T cell-induced CRS [7]. Third, many patients received concomitant therapies that could impact clinical outcomes. Fourth, many of our respiratory infections were diagnosed based on tracheal aspirates because bronchoscopies were infrequent at the time. Lastly, the study was descriptive and not aimed to investigate predisposing risk factors for infectious complications or to determine tocilizumab efficacy.

## Conclusion

In this study, we demonstrated the effects of tocilizumab in 60 patients with COVID-19. We primarily used tocilizumab in patients presenting with signs of cytokine release syndrome and acute respiratory distress syndrome. Many patients achieved clinical improvement and were eventually discharged from the hospital. Our most interesting finding was the rebound effect seen with C-reactive protein after day 10, which suggests the need for higher or subsequent doses. Similar to prior studies, infectious complications after tocilizumab were not uncommon. Our results highlight the need for more robust studies investigating the safety, efficacy, and optimal timing of tocilizumab in COVID-19 patients.

## Supplementary Information

**Additional file 1: Appendix 1.** Patients meeting institution-specific tocilizumab criteria. **Appendix 2.** Clinical measures within 14 days of receiving tocilizumab. **Appendix 3.** Infectious complications within 30 days of receiving tocilizumab

## Data Availability

The datasets used in this study are available from the corresponding author on reasonable request.

## Abbreviations

COVID-19: Coronavirus Disease 2019
SARS-CoV-2: Severe acute respiratory syndrome coronavirus 2
CRS: Cytokine release syndrome
IL-6: Interleukin-6
ARDS: Acute respiratory distress syndrome
CAR: Chimeric antigen receptor
IV: Intravenous
SpO2: Saturation of peripheral oxygen
PCR: Polymerase chain reaction
WHO: World Health Organization
ICU: Intensive care unit
PaO_2_/FiO_2_: Partial pressure of oxygen/fraction of inspired oxygen
ABG: Arterial blood gas
AST: Aspartate aminotransferase
CRP: C-reactive protein
ESR: Erythrocyte sedimentation rate
LDH: Lactate dehydrogenase
NLR: Neutrophil-to-lymphocyte ratio

## References

[1] Chen N, Zhou M, Dong X, Qu J, Gong F, Han Y, Qiu Y, Wang J, Liu Y, Wei Y, Xia J, Yu T, Zhang X, Zhang L (2020). Epidemiological and clinical characteristics of 99 cases of 2019 novel coronavirus pneumonia in Wuhan, China: a descriptive study. Lancet.

[2] Ackermann M, Verleden SE, Kuehnel M, Haverich A, Welte T, Laenger F, Vanstapel A, Werlein C, Stark H, Tzankov A, Li WW, Li VW, Mentzer SJ, Jonigk D. Pulmonary vascular endothelialitis, thrombosis, and angiogenesis in Covid-19. N Engl J Med. 2020;383:120–8. 10.1056/NEJMoa2015432. 10.1056/NEJMoa2015432 PMC7412750 32437596

[3] Brune K, Frank J, Schwingshackl A, Finigan J, Sidhaye VK (2015). Pulmonary epithelial barrier function: some new players and mechanisms. Am J Physiol Lung Cell Mol Physiol.

[4] Siddiqi HK, Mehra MR (2020). COVID-19 illness in native and immunosuppressed states: a clinical-therapeutic staging proposal. J Heart Lung Transplant.

[5] Zhang C, Wu Z, Li J, Zhao H, Wang G (2020). Cytokine release syndrome in severe COVID-19: interleukin-6 receptor antagonist tocilizumab may be the key to reduce the mortality. Int J Antimicrob Agents.

[6] Coomes EA and Haghbayan H. Interleukin-6 in COVID-19: A systematic review and meta-analysis. medRxiv 2020. 10.1101/2020.03.30.20048058. (pre-print). 10.1002/rmv.2141 PMC7460877 32845568

[7] Le RQ, Li L, Yuan W, Shord SS, Nie L, Habtemariam BA, Przepiorka D, Farrell AT, Pazdur R (2018). FDA approval summary: tocilizumab for treatment of chimeric antigen receptor t cell-induced severe or life-threatening cytokine release syndrome. Oncologist.

[8] Giamarellos-Bourboulis EJ, Netea MG, Rovina N, Akinosoglou K, Antoniadou A, Antonakos N, Damoraki G, Gkavogianni T, Adami M, Katsaounou P, Ntaganou M, Kyriakopoulou M, Dimopoulos G, Koutsodimitropoulos I, Velissaris D, Koufargyris P, Karageorgos A, Katrini K, Lekakis V, Lupse M, Kotsaki A, Renieris G, Theodoulou D, Panou V, Koukaki E, Koulouris N, Gogos C, Koutsoukou A. Complex Immune Dysregulation in COVID-19 Patients with Severe Respiratory Failure. Cell Host Microbe 2020; 27(6):992–1000.e3. 10.1016/j.chom.2020.04.009 PMC7172841 32320677

[9] Alzghari SK, Acuña VS (2020). Supportive treatment with tocilizumab for COVID-19: a systematic review. J Clin Virol.

[10] Luo P, Liu Y, Qiu L, Lui X, Liu D, Li J (2020). Tocilizumab treatment in COVID-19: a single center experience. J Med Virol.

[11] Xu X, Han M, Li T, Sun W, Wang D, Fu B, Zhou Y, Zheng X, Yang Y, Li X, Zhang X, Pan A, Wei H (2020). Effective treatment of severe COVID-19 patients with Tocilizumab. Proc Natl Acad Sci U S A.

[12] Sciascia S, Aprà F, Baffa A, Baldovino S, Boaro D, Boero R, Bonora S, Calcagno A, Cecchi I, Cinnirella G, Converso M, Cozzi M, Crosasso P, De Iaco F, Perri GD, Eandi M, Fenoglio R, Giusti M, Imperiale D, Imperiale G, Livigni S, Manno E, Massara C, Milone V, Natale G, Navarra M, Oddone V, Osella S, Piccioni P, Radin M, Roccatello D, Rossi D (2020). Pilot prospective open, single arm multicentre study on off-label use of tocilizumab in patients with severe COVID-19. Clin Exp Rheumatol.

[13] Capra R, De Rossi N, Mattioli F, Romanelli G, Scarpazza C, Sormani MP, Cossi S (2020). Impact of low dose tocilizumab on mortality rate in patients with COVID-19 related pneumonia. Eur J Intern Med.

[14] Hermine O, Mariette X, Tharaux P, Resche-Rigon M, Porcher R, Ravaud P. CORIMUNO-19 Collaborative Group. JAMA Intern Med. 2020. 10.1001/jamainternmed.2020.6820.

[15] Somers EC, Eschenauer GA, Troost JP, Golob JL, Gandhi TN, Wang L, Zhou N, Petty LA, Baang JH, Dollman NO, Frame D, Gregg KS, Kaul DR, Nagel J, Patel TS, Zhou S, Lauring AS, Hanauer DA, Martin E, Sharma P, Fung CM, Pogue JM. Tocilizumab for treatment of mechanically ventilated patients with COVID-19. medRxiv 2020. 10.1101/2020.05.29.20117358. (preprint). 10.1093/cid/ciaa954 PMC7454462 32651997

[16] Guaraldi G, Meschiari M, Cozzi-Lepri A, Milic J, Tonelli R, Menozzi M, Franceschini E, Cuomo G, Orlando G, Borghi V, Santoro A, Di Gaetano M, Puzzolante C, Carli F, Bedini A, Corradi L, Fantini R, Castaniere I, Tabbì L, Girardis M, Tedeschi S, Gianella M, Bartoletti M, Pascale R, Dolci G, Brugiono L, Pietrangelo A, Cossarizza A, Pea F, Clini E, Salvarani C, Massari M, Viale PL, Mussini C (2020). Tocilizumab in patients with severe COVID-10: a retrospective cohort study. Lancet Rhematol.

[17] Biran N, Ip A, Ahn J, Go RC, Wang S, Mathura S, Sinclaire BA, Bednarz U, Marafelias M, Hansen E, Siegel DS, Goy AH, Pecora AL, Sawczuk IS, Koniaris LS, Simwenyi M, Varga DW, Tank LK, Stein AA, Allusson V, Lin GS, Oser WF, Tuma RA, Reichman J, Brusco L, Carpenter KL, Costanzo EJ, Vivona V, Goldberg SL (2020). Tocilizumab among patients with COVID-19 in the intensive care unit: a multicentre observational study. Lancet Rhematol.

[18] Salvarani C, Dolci G, Massari M, Merlo DF, Cavuto S, Savoldi L, Bruzzi P, Boni F, Braglia L, Turrà C, Ballerini PF, Sciascia R, Zammarchi L, Para O, Scotton PG, Inojosa WO, Ravagnani V, Salerno ND, Sainaghi PP, Brignone A, Codeluppi M, Teopompi E, Milesi M, Bertomoro P, Claudio N, Salio M, Falcone M, Cenderello G, Donghi L, Del Bono V, Colombelli PL, Angheben A, Passaro A, Secondo G, Pascale R, Piazza I, Facciolongo N, Costantini M, RCT-TCZ-COVID-19 Study Group. Effect of tocilizumab vs standard care on clinical worsening in patients hospitalized with COVID-19 pneumonia. JAMA Intern Med. 2020. 10.1001/jamainternmed.2020.6615.

[19] Martínez-Sanz J, Muriel A, Ron R, Herrera S, Pérez-Molina JA, Moreno S, Serrano-Villar S. Effects of tocilizumab on mortality in hospitalized patients with COVID-19: a multicenter cohort study. Clin Microbiol Infect. 2020. 10.1016/j.cmi.2020.09.021. 10.1016/j.cmi.2020.09.021 PMC7510451 32979572

[20] Gupta S, Wang W, Hayek SS, Chan L, Mathews KS, Melamed ML, Brenner SK, Leonberg-Yoo A, Schenck EJ, Radbel J, Reiser J, Bansal A, Srivastava A, Zhou Y, Finkel D, Green A, Mallappallil M, Faugno AJ, Zhang J, Velez JC, Shaefi S, Parikh CR, Charytan DM, Athavale AM, Friedman AN, Redfern RE, Short SAP, Correa S, Pokharel KK, Admon AJ, Donnelly JP, Gershengorn HB, Douin DJ, Semler MW, Hernán MA, Leaf DE. STOP-COVID investigators. JAMA Intern Med. 2020. 10.1001/jamainternmed.2020.6252.

[21] Stone JH, Frigault MJ, Serling-Boyd NJ, Fernandes AD, Harvey L, Foulkes AS, Horick NK, Healy BC, Shah R, Bensaci AM, Wooley AE, Nikiforow S, Lin N, Sagar M, Schrager H, Huckins DS, Axelrod M, Pincus MD, Fleisher J, Sacks CA, Dougan M, North CM, Halvorsen Y, Thurber TK, Dagher Z, Scherer A, Wallwork RS, Kim AY, Schoenfeld S, Sen P, Neilan TG, Perugino CA, Unizony SH, Collier DS, Matza MA, Yinh JM, Bowman KA, Meyerowitz E, Zafar A, Drobni ZD, Bolster MB, Kohler M, KM D’S, Dau J, Lockwood MM, Cubbison C, Weber BN, Mansour MK, BACC Bay Tocilizumab Trial Investigators. Efficacy of tocilizumab in patients hospitalized with Covid-19. N Engl J Med. 2020;383:2333–44. 10.1056/NEJMoa2028836. 10.1056/NEJMoa2028836 PMC7646626 33085857

[22] China's National Health Commission. Novel coronavirus treatment guidelines – 7th Edition. 2020.

[23] World Health Organization. WHO R&D Blueprint novel Coronavirus: COVID-19 Therapeutic Trial Synopsis. 2020.

[24] Rice TW, Wheeler AP, Bernard GR, Hayden DL, Schoenfeld DA, Ware LB (2007). Comparison of the SpO2/FIO2 ratio and the PaO2/FIO2 ratio in patients with acute lung injury or ARDS. Chest.

[25] Definition Task Force ARDS, Ranieri VM, Rubenfeld GD, Thompson BT, Ferguson ND, Caldwell E, Fan E, Camporota L, Slutsky AS (2012). Acute respiratory distress syndrome: the Berlin Definition. JAMA.

[26] ClinicalTrials.gov. A Study to Evaluate the Efficacy and Safety of Tocilizumab in Hospitalized Participants With COVID-19 Pneumonia (EMPACTA). Available from https://clinicaltrials.gov/ct2/show/NCT04372186. Accessed 27 Sep 2020.

[27] Lippi G, Plebani M (2020). Laboratory abnormality in patients with COVID-2019 infection. Clin Chem Lab Med.

[28] Actemra (tocilizumab) [prescribing information]. South San Francisco: Genentech Inc; 2020.

[29] Nishimoto N, Terao K, Mima T, Nakahara H, Takagi N, Kakehi T (2008). Mechanisms and pathologic significance in increase in serum interleukin-6 (IL-6) and soluble IL-6 receptor after administration of an anti-IL-6 receptor antibody, tocilizumab, in patients with rheumatoid arthritis and Castleman disease. Blood.

[30] Rimland CA, Morgan CE, Bell GJ, Kim MK, Hedrick T, Marx A, Bramson B, Swygard H, Napravnik S, Schmitz JL, Carson SS, Fischer WA, Eron JJ, Gay CL, Parr JB. Clinical characteristics and early outcomes in patients with COVID-19 treated with tocilizumab at a United States academic center. medRxiv 2020. 10.1101/2020.05.13.20100404. (preprint).

[31] Campochiaro C, Della-Torre E, Cavalli G, De Luca G, Ripa M, Boffini N, Tomelleri A, Baldissera E, Rovere-Querini P, Ruggeri A, Monti G, De Cobelli F, Zangrillo A, Tresoldi M, Castagna A, Dagna L, TOCI-RAF Study Group (2020). Efficacy and safety of tocilizumab in severe in COVID-19 patients: a single-Centre retrospective cohort study. Eur J Intern Med.

[32] Horby P, Lim WS, Emberson JR, Mafham M, Bell JL, Linsell L, Staplin N, Brightling C, Ustianowski A, Elmahi E, Prudon B, Green C, Felton T, Chadwick D, Rege K, Fegan C, Chappell LC, Faust SN, Jaki T, Jeffery K, Montgomery A, Rowan K, Juszczak E, Baillie JK, Haynes R, Landray MJ. RECOVERY Collaborative Group. N Engl J Med. 2020. 10.1056/NEJMoa2021436.

[33] Navarro G, Taroumian S, Barroso N, Duan L, Furst D (2014). Tocilizumab in rheumatoid arthritis: a meta-analysis of efficacy and selected clinical outcomes. Semin Arthritis Rheum.

[34] Hill JA, Li D, Hay KA, Green ML, Cherian S, Chen X, Riddell SR, Maloney DG, Boeckh M, Turtle CJ (2018). Infectious complications of CD19-targeted chimeric antigen receptor-modified T-cell immunotherapy. Blood.

[35] Kimmig LM, Wu D, Gold M, Pettit NN, Pitrak D, Mueller J, Husain AN, Mutlu EA, Mutlu GM. IL6 inhibition in critically ill COVID-19 patients is associated with increased secondary infections. medRxiv 2020. 10.1101/2020.05.15.20103531. (preprint). 10.3389/fmed.2020.583897 PMC7655919 33195334

