# Supplementary material for: Effects of Tocilizumab in COVID-19 patients: a cohort study
Source: BMC Infect Dis. 2020 Dec 22;20:964. doi: 10.1186/s12879-020-05701-4 (PMC7755065; doi:10.1186/s12879-020-05701-4)
Supplement: Supplementary file 1 — Additional file 1: Appendix 1. Patients meeting institution-specific tocilizumab criteria. Appendix 2. Clinical measures within 14 days of receiving tocilizumab. Appendix 3. Infectious complications within 30 days of receiving tocilizumab [file 12879_2020_5701_MOESM1_ESM.docx]

**SUPPLEMENTAL MATERIAL**

Appendix 1. Patients meeting institution-specific tocilizumab criteria

| All of the following must be met: | n (%) | Number of patients with available data |
| --- | --- | --- |
| Confirmed or highly suspected COVID-19 | 60 (100.0) | 60 |
| Requiring minimum of 4 liters nasal cannula | 56^a^ (93.3) | 60 |
| Clinical deterioration based on physician discretion | 60 (100.0) | 60 |
| At least 2 of the following:  Interleukin-6 >40 pg/mL  C-reactive protein >10 mg/dL  Lactate dehydrogenase >350 U/L  Ferritin >1000 ng/mL  D-dimer >1 mcg/mL | 33 (75.0)  50 (83.3)  52 (92.9)  36 (64.3)  37 (64.9) | 44  60  56  56  57 |

^a^Four patients received tocilizumab but did not meet criteria: room air (1), 2 liters nasal cannula (3)

Appendix 2. Clinical measures within 14 days of receiving tocilizumab

| n (%) | All patients (N=60) | Died (n=9) | Alive (n=51) | Discharged (n=22) | Still Hospitalized (n=29) | ICU  (n=52) | Floor  (n=8) | Invasive mechanical ventilation (n=29) | No invasive mechanical ventilation (n=31) |
| --- | --- | --- | --- | --- | --- | --- | --- | --- | --- |
| Renal complications  Acute kidney injury^a^  Renal replacement therapy^b^ | 33 (55.0)  12 (20.0) | 9 (100.0)  3 (33.3) | 24 (47.1)  9 (17.6) | 4 (18.2)  0 (0.0) | 20 (69.0)  9 (31.0) | 31 (59.6)  12 (23.1) | 2 (25.0)  0 (0.0) | 24 (82.8)  12 (41.4) | 9 (29.0)  0 (0.0) |
| Liver complications  Transaminitis^c^  Bilirubinemia^d^ | 14 (23.3)  10 (16.7) | 4 (44.4)  4 (44.4) | 10 (19.6)  6 (11.8) | 4 (18.2)  2 (9.1) | 6 (20.7)  4 (13.8) | 10 (19.2)  8 (15.4) | 4 (50.0)  2 (25.0) | 7 (24.1)  6 (20.7) | 7 (22.6)  4 (12.9) |
| Cardiovascular complications  Vasopressor use | 24 (40.0) | 5 (55.6) | 19 (37.3) | 1 (4.5) | 18 (62.1) | 24 (46.2) | 0 (0.0) | 23 (79.3) | 1 (3.2) |
| Respiratory complications  ARDS, n (%)^e^  Mild (201<PaO_2_/FiO_2_≤300)  Moderate (101<PaO_2_/FiO_2_≤200)  Severe (0<PaO_2_/FiO_2_ ≤100)  Proning  ECMO  Paralytics  Inhaled nitric oxide | 4 (6.7)  20 (33.3)  33 (55.0)  24 (40.0)  3 (5.0)  19 (31.7)  5 (8.3) | 0 (0.0)  2 (22.2)  7 (77.8)  6 (66.7)  0 (0.0)  4 (44.4)  1 (11.1) | 4 (7.8)  18 (35.3)  26 (51.0)  18 (35.3)  3 (5.9)  15 (29.4)  4 (7.8) | 4 (18.2)  9 (40.9)  6 (27.3)  4 (18.2)  0 (0.0)  0 (0.0)  0 (0.0) | 0 (0.0)  9 (31.0)  20 (69.0)  14 (48.3)  3 (10.3)  15 (51.7)  4 (13.8) | 2 (3.8)  18 (34.6)  31 (59.6)  23 (44.2)  3 (5.8)  19 (36.5)  5 (9.6) | 2 (25.0)  2 (25.0)  2 (25.0)  1 (12.5)  0 (0.0)  0 (0.0)  0 (0.0) | 0 (0.0)  12 (41.4)  17 (58.6)  16 (55.2)  3 (10.3)  19 (65.5)  4 (13.8) | 4 (12.9)  8 (25.8)  16 (51.6)  8 (25.8)  0 (0.0)  0 (0.0)  1 (3.2) |
| Mode of oxygen supplementation^f^  Nasal cannula  Venti-mask  Nonrebreather  High-flow nasal cannula  BiPAP  Invasive mechanical ventilation | 12 (20.0)  0 (0.0)  3 (5.0)  14 (23.3)  2 (3.3)  29 (48.3) | 0 (0.0)  --  1 (11.1)  2 (22.2)  0 (0.0)  6 (66.7) | 12 (23.5)  --  2 (3.9)  12 (23.5)  2 (3.9)  23 (45.1) | 12/22  --  1/22  7/22  1 (4.5)  1 (4.5) | 0 (0.0)  --  1 (3.4)  5 (17.2)  1 (3.4)  22 (75.9) | 6 (11.5)  --  2 (3.8)  13 (25.0)  2 (3.8)  29 (55.8) | 6 (75.0)  --  1 (12.5)  1 (12.5)  0 (0.0)  0 (0.0) | --  --  --  --  --  -- | --  --  --  --  --  -- |
| SOFA score, median (range)^g^ | 4 (0-14) | 6 (4-11) | 3 (0-14) | 2 (0-4) | 6 (1-14) | 4.5 (0-14) | 2 (1-4) | 8 (2-14) | 2.5 (0-10) |

^a^Serum creatinine increase ≥0.3 mg/dL within 48 hours or increase by 50% from baseline

^b^3 hemodialysis, 9 continuous veno-venous hemofiltration (CVVH)

^c^ALT/AST > 5x ULN

^d^Total bilirubin >1.3 mg/dL

^e^Categorized by lowest PaO_2_/FiO_2_ ratio

^f^Categorized by highest degree of oxygen support

^g^Categorized by highest SOFA score

Appendix 3. Infectious complications within 30 days of receiving tocilizumab (N=60)

|  | Source | Pathogen identified | Time after administration (days) | Concomitant immunosuppressants |
| --- | --- | --- | --- | --- |
| Patient 1 | Blood | MRSA | 28 | Corticosteroids |
| Patient 2 | Tracheal Aspirate | MRSA | 9 | Corticosteroids |
|  | Tracheal Aspirate | MSSA | 28 |  |
|  | Blood | *Klebsiella pneumoniae* | 30 |  |
| Patient 3 | Blood | Diptheroids | 11 | Corticosteroids |
|  | Blood | Diptheroids, *Enterococcus faecalis* | 13 |  |
|  | Blood | *Enterococcus faecalis* | 24 |  |
| Patient 4 | Blood | *Serratia marcescens* | 15 | Corticosteroids |
|  | Tracheal Aspirate | *Serratia marcescens* | 15 |  |
| Patient 5 | Tracheal Aspirate | MSSA | 5 | None |
| Patient 6 | Bronchial aspirate | *Pseudomonas aeruginosa* | 13 | Corticosteroids |
|  | Blood | *Staphylococcus capitis* | 21 |  |
|  | Tracheal Aspirate | *Pseudomonas aeruginosa, Stenotrophomonas maltophilia* | 23 |  |
| Patient 7 | Tracheal Aspirate | MRSA, *Pseudomonas aeruginosa* | 24 | Corticosteroids |
| Patient 8 | Blood | *Enterococcus faecalis* | 2 | Corticosteroids |
|  | Blood | *Candida glabrata* | 4 |  |
| Patient 9 | Tracheal Aspirate | *Escherichia coli* | 8 | Corticosteroids |
|  | Tracheal Aspirate | *Klebsiella pneumoniae* | 15 |  |
|  | Tracheal Aspirate | MSSA | 22 |  |
| Patient 10 | Blood | *Staphylococcus epidermidis* | 24 | None |
| Patient 11 | Urine | *Escherichia coli* | 18 | None |
|  | Blood | *Staphylococcus epidermidis* | 22 |  |
| Patient 12 | Endotracheal | *Klebsiella aerogenes* | 2 | None |
|  | Tracheal Aspirate | *Klebsiella aerogenes* | 24 |  |
| Patient 13 | Sputum Induced | *Klebsiella aerogenes* | 6 | None |
| Patient 14 | Blood | *Staphylococcus epidermidis* | 17 | Corticosteroids |
| Patient 15 | Blood | *Staphylococcus hominis* | 9 | None |
| Patient 16 | Blood | *Staphylococcus epidermidis* | 10 | Corticosteroids |
|  | Sputum Expectorated | *Klebsiella aerogenes* | 10 |  |

MRSA methicillin-resistant *Staphylococcus aureus*; MSSA methicillin-sensitive *Staphylococcus aureus*
